# Self-Organized Plasmonic Nanowire Arrays Coated with Ultrathin TiO_2_ Films for Photoelectrochemical Energy Storage

**DOI:** 10.1021/acsanm.3c03546

**Published:** 2023-11-15

**Authors:** Maria Caterina Giordano, Long Duy Pham, Giulio Ferrando, Hieu Si Nguyen, Chi Ha Le, The-Hung Mai, Giorgio Zambito, Matteo Gardella, Francesco Buatier de Mongeot

**Affiliations:** † Dipartimento di Fisica, Università di Genova, Via Dodecaneso 33, 16146 Genova, Italy; ‡ Institute of Materials Science, Vietnam Academy of Science and Technology, 18 Hoang Quoc Viet 11300, Cau Giay, Hanoi, Vietnam

**Keywords:** large-area nanoelectrodes, Au nanowires, ultrathin TiO_2_ films, plasmonic nanoantennas, hot-electrons, photoelectrochemical devices, energy storage

## Abstract

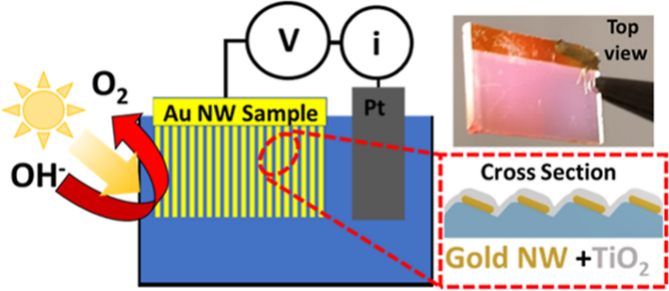

The strategic field of renewable energy production and storage requires novel nanoscale platforms that can feature competitive solar energy conversion properties. Photochemical reactions that promote energy storage, such as water splitting and oxygen–hydrogen evolution reactions, play a crucial role in this context. Here, we demonstrate a novel photoelectrochemical device based on large-area (cm^2^) self-organized Au nanowire (NW) arrays, uniformly coated with ultrathin TiO_2_ films. The NW arrays act both as transparent nanoelectrodes and as a plasmonic metasurface that resonantly enhances the very weak visible photocurrent generated by a prototype photoelectrochemical oxygen evolution reaction. We demonstrate a polarization-sensitive plasmon-enhanced photocurrent that reaches a gain of about 3.8 in the visible spectral range. This highlights the potential of our novel nanopatterned plasmonic platform in photochemistry and energy storage.

## Introduction

The continuous increase in energy consumption and the dramatic consequences of global climate change urgently demand effective strategies for renewable energy conversion. However, the need for nonpolluting energy storage strategies, that can be easily integrated over a large scale, currently represents a crucial issue for the effective use and integration of renewable energy sources that are intrinsically intermittent (e.g., sun and wind).^1^ Photochemical reactions, such as photocatalysis and water splitting, are receiving special attention from the scientific community since they can promote molecular degradation of organic pollutants, or energy storage via hydrogen production in view of renewable energy conversion.^2−11^ Even though different hydrogen production schemes have been investigated, many challenges still need to be addressed. Among photoelectrochemical materials endowed with strong photocatalytic activity, chemical stability, and suitable electronic structure to promote O_2_ and H_2_ generation reactions, we can mention the family of large band gap semiconductor oxides (TiO_2_, ZnO, and WO_3_) that only absorb a limited portion (5%) of the solar light in the ultraviolet (UV) spectrum.^12,13^ Conversely, narrow band gap semiconductors, capable of absorbing a wide range of solar light in the visible spectrum, are typically limited in terms of their chemical reactivity, selectivity, and/or photochemical stability.^14^ In addition, the high recombination rate of these materials significantly dampens their energy conversion efficiency.^15^

So far, intense research efforts have been focused on overcoming these limitations to improve the performance of photocatalytic electrodes. Doping strategies have, e.g., been adopted to reduce the band gap of photocatalytic materials,^16^ while heterojunctions have been fabricated to increase the separation rate of photoexcited carriers.^17,18^ A promising approach is based on the excitation of localized plasmon resonances in nanoparticles to promote photoconversion efficiency and to control the selectivity in photocatalysis.^19−24^ Under this condition, the ultrafast temporal dynamics and decay of plasmonic “hot-electrons” have recently attracted considerable attention as an effective carrier injection channel in photocatalysis.^25−29^ In parallel, the characteristic plasmonic near-field confinement has been shown to play a relevant role in these processes.^30^ Very recently, novel strategies and materials have also been investigated to exploit light-induced photothermal effects in catalysis without the need for extreme concentration to achieve high temperatures.^31,32^

State-of-the-art photoelectrochemical devices typically rely on thin transparent conductive oxide (TCO) electrodes that support the active photocatalyst.^33−35^ Conductive oxide films represent a diffuse material platform in optoelectronics but are based on scarce and polluting elements, such as indium, tin, or fluorine, that give rise to environmental issues when used in large-scale applications. TCOs also need high-temperature treatments to improve their relatively poor electronic transport properties and typically suffer from severe limitations due to brittleness and delamination problems in the thin film configuration. These factors strongly limit their use in new-generation flexible devices and stimulate the development of alternative nanoscale conductive materials.^36−39^

In this work, we demonstrate a large-area photochemical device based on self-organized nanoelectrodes acting both as a transparent conductive layer and as a plasmonic metasurface that can resonantly boost the efficiency of a reference photoelectrochemical reaction such as the oxygen evolution reaction of a NaOH solution. Self-organized nanoarrays based on out-of-plane tilted nanostrips are fabricated over a large area (cm^2^) by combining ion-assisted nanopatterning of dielectric templates with a glancing angle metal evaporation. The approach allows for effective control of the electronic transport properties of the nanoarrays combined with additional plasmonic functionalities. This way, a hybrid plasmonic–oxide metasurface can be engineered by decorating the nanoantenna arrays with thin TiO_2_ films. Under this configuration, we demonstrate broadband amplification of the photocurrent induced in a nanopatterned device with respect to a flat reference sample, detected over the near-UV to the visible spectral range. A spectrally selective detection of the photocurrent additionally shows a plasmon-enhanced effect in the visible spectral range, when the plasmonic nanoarrays capped by an ultrathin oxide film are coupled in close proximity to the solution.

## Results and Discussion

**Figure 1:**
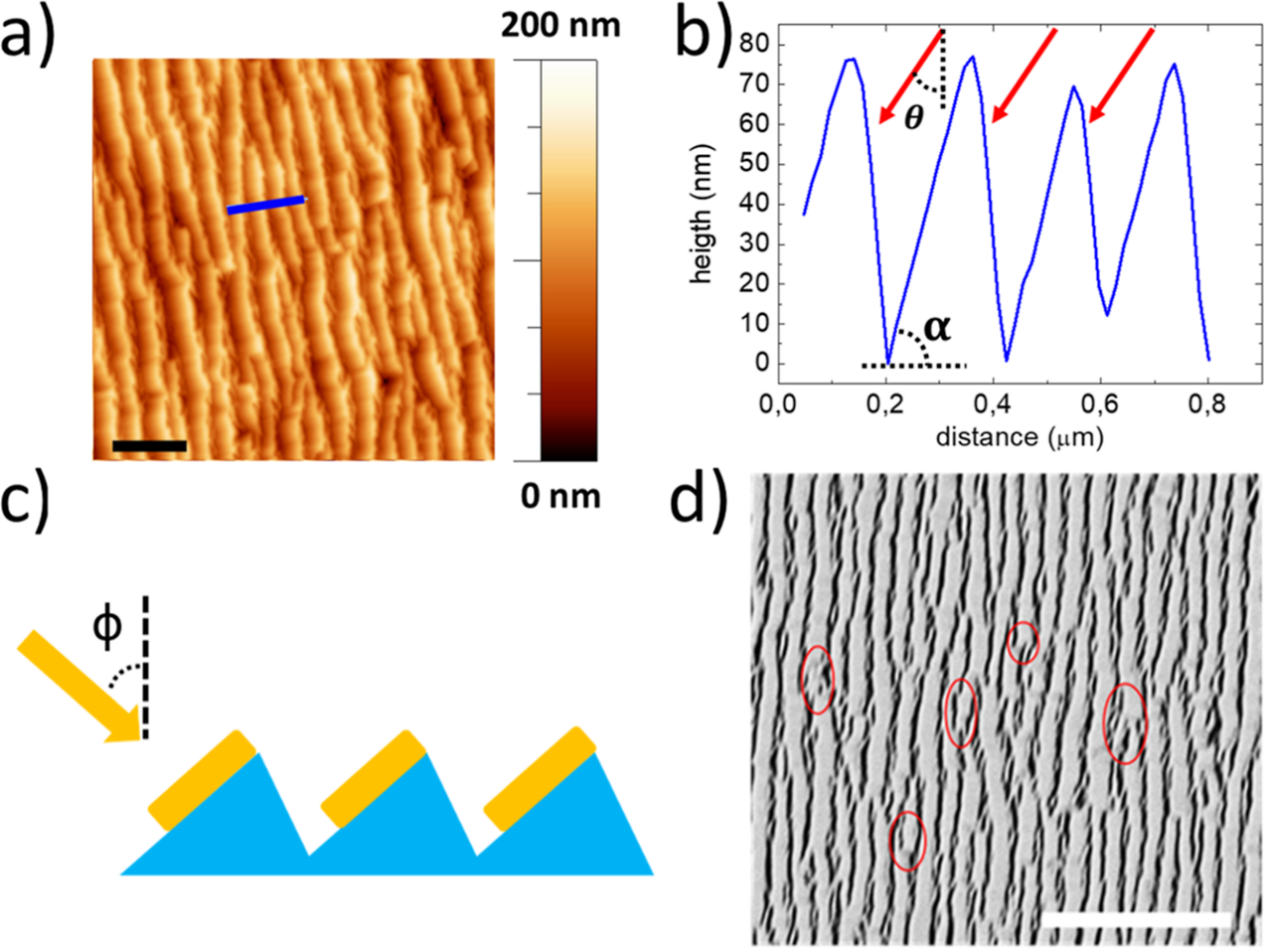
(a,b) AFM topography of the quasi-1D nanopatterned glass template and the corresponding cross-section profile (blue line in panel a). (c,d) Sketch of the Au glancing angle evaporation onto the faceted templates and scanning electron microscopy (SEM) image (backscattered electron signal) of the self-organized Au nanowire arrays. The white scale bar corresponds to 2 μm and the red circles highlight the lateral interconnections between the nanowires.

Large-area nanopatterns are fabricated onto a low-cost soda-lime glass by using a self-organized nanopatterning process. Here, a low-cost soda-lime glass wafer is exposed to an Ar ion beam (energy of 800 eV) impinging at the incidence angle θ = 30° with respect to the surface normal, while the substrate is heated and kept at a temperature of about 680 K, close to its transition point. The self-organized process exploits an ion-assisted wrinkling instability arising under ion beam irradiation of dielectric substrates,^40^ which allows for obtaining a quasi-one-dimensional (1D) nanopattern that spreads uniformly all over the cm^2^ glass surface (Figure 1a). The periodic nanoripples with a long axis running orthogonal to the ion beam projection are characterized by a vertical dynamic as high as 80–100 nm, as shown in the AFM cross-section profile (Figure 1b), and by a lateral periodicity of about 260 nm, as calculated by the autocorrelation of the AFM image (see the Supporting Information, Figure S1). Remarkably, the periodic nanoripples show a peculiar asymmetric profile characterized by slope-selected nanofacets, whose local tilt can be easily controlled by tailoring the ion beam irradiation conditions.^41^ In this work, we engineer the glass template morphology to achieve an asymmetric sawtooth profile with the facets opposing the ion beam direction peaked at about α = 30° (Figure 1b).

Thanks to the faceted morphology of the glass templates, large-area quasi-1D nanostructure arrays of an arbitrary material can be precisely confined in a single maskless-lithography step by exploiting the shadowing effect of the periodic ridges under glancing angle thermal evaporation conditions, as sketched in Figure 1c. In the present case, we performed gold evaporation at ϕ = 60° with respect to the surface normal direction, orienting the sample so that the ordered nanofacets of the glass template tilted at α = 30° face the Au beam during thermal evaporation under ultra high vacuum (UHV) conditions. In this way, it is possible to engineer semitransparent Au nanowire (NW) electrodes to develop large-area conductive electrodes for photoelectrochemical applications. In addition, the template prepared following the self-organized approach allows, by simply exploiting the geometrical shadowing, the deposit of alternative materials to gold, such as copper and platinum.

The SEM image of Figure 1d, acquired with the backscattered electron detector, reveals the lateral distribution of the self-organized array of Au NWs, tilted with respect to the sample plane and laterally disconnected by the shadowed facets. The maskless lithographic approach additionally enables to control accurately the shape of the metallic NWs, namely, their width/height aspect ratio, by tailoring the metal evaporation angle ϕ, which defines the NW width *w*, and the evaporation dose, which defines the NW thickness *h*. Under our experimental conditions, the Au NWs are endowed with lateral width *w* = 100 nm and local thickness *h* = 50 nm, as determined by a statistical analysis of the SEM image and measurement of the deposited Au film thickness using a calibrated quartz microbalance.

**Figure 2:**
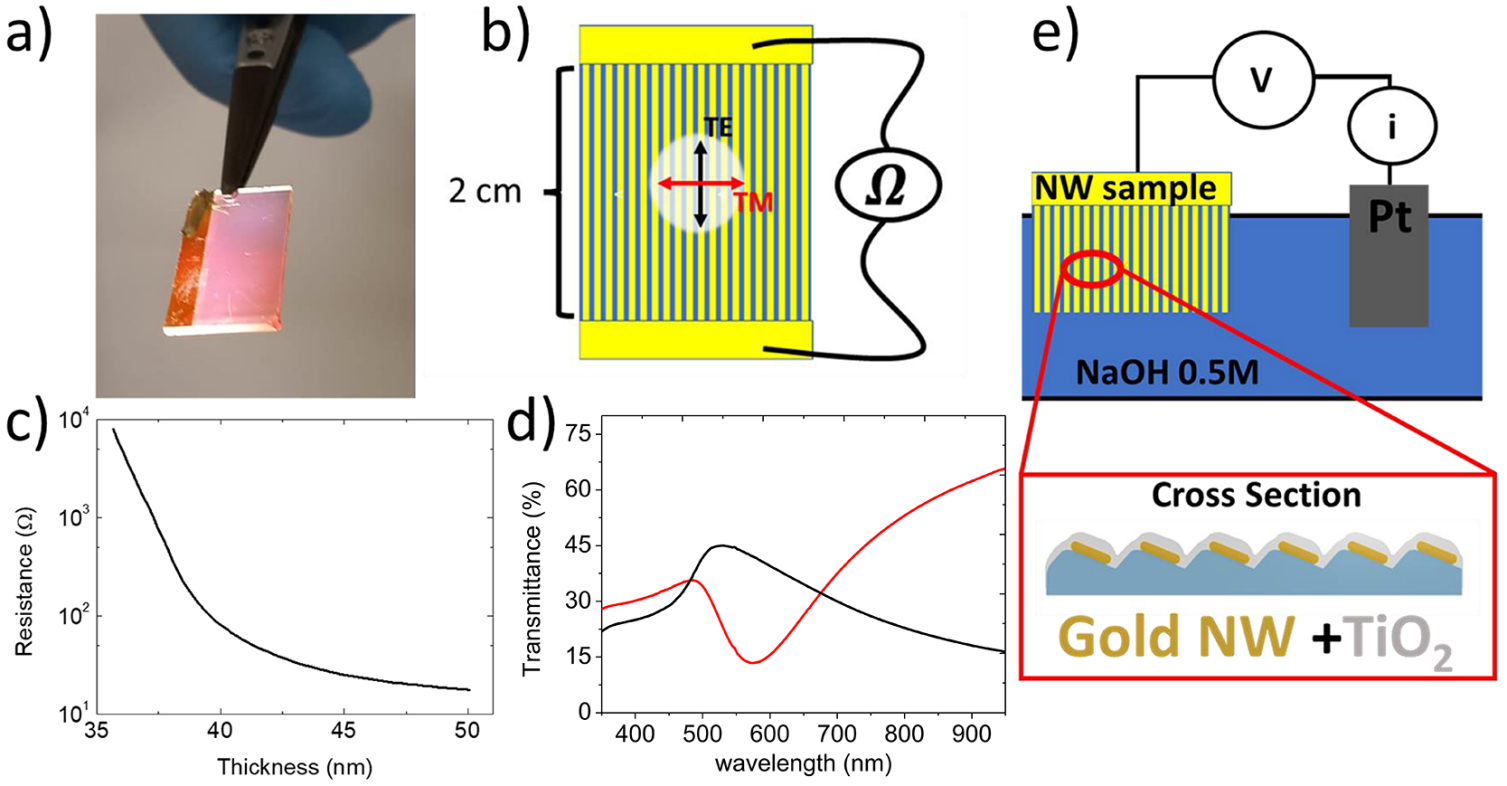
(a,b) Picture of the large-area plasmonic nanoarrays featuring enhanced light scattering and sketch of the configuration used for the in situ electrical transport characterization, respectively. Two-wire sheet resistance measurements have been performed in situ parallel to the long axis of the NW arrays with two electrodes facing each other at a 2 cm distance. (c) Longitudinal sheet resistance plotted as a function of the local Au thickness (h) deposited on top of the exposed facets. (d) Optical transmission of the Au NW arrays detected for longitudinal (TE—black line) and transversal (TM—red line) polarization of the incident beam with respect to the NW long axis, as sketched in panel b. (e) Schematic illustration of the system (structure/chemical composition) and interconnection to the electrochemical cell.

The photograph in Figure 2a shows the nanopatterned sample, which is coated with Au NW arrays over large areas in the range of cm^2^ required for the photocatalysis experiments. The semitransparent NW sample is illuminated from the bottom by an unpolarized halogen lamp, and the reddish color is determined by scattering from the Au NWs, which act as plasmonic nanoantennas.^42^

The geometry of the faceted template and the shadow deposition conditions employed here promote the formation of a percolated NW network when contact electrodes are placed in the axial direction parallel to the NW axis, as shown in the sketch of Figure 2b. The electrical transport properties of the NW electrodes have been monitored in situ during deposition to optimize their sheet resistance, exploiting two macroscopic Ti/Au electrodes placed at a distance of 2 cm in the direction of the NW axis.

As shown in Figure 2c, which plots the longitudinal sheet resistance of the Au NW electrode as a function of deposited Au thickness, we obtain resistance values in the range of 10 kΩ/□ for a thickness of the Au NWs of about 35 nm. By increasing their thickness by just 5 nm, up to 40 nm, we observe a dramatic resistance drop by more than 2 orders of magnitude down to 77 Ω/□. We attribute this to the formation of a densely percolated network, when lateral interconnections between the NWs are formed in correspondence to the dislocations of the self-organized pattern (highlighted by the representative red circles in the SEM image in Figure 1d). To further optimize the electrical transport properties of the Au NW template, we increase the gold NW thickness up to 50 nm, thus reaching a sheet resistance value as low as 17.6 Ω/□, which is competitive with the best TCOs.^43^ Under these conditions, the sample is highly conductive, while in the transverse direction, the Au NWs are still laterally disconnected, as shown by the SEM image of the sample (Figure 1d). Thanks to this peculiar anisotropic nanoscale morphology, strong optical dichroism has been detected by far-field optical transmission spectroscopy (Figure 2d) performed by illuminating the sample at normal incidence with a broadband (near-UV–visible—near-IR) polarized optical beam. For light polarization parallel to the longitudinal axis of the NWs (TE-pol, black line in Figure 2d), the NW arrays behave as a thin compact film, and their optical transmission spectrum is characterized by a local maximum at about 500 nm, corresponding to the onset of Au interband transitions. Conversely, for light polarization transverse to the NW axis (TM-pol, red line in Figure 2d), a broadband transmission minimum centered at 580 nm wavelength is detected, due to excitation of localized surface plasmon resonance (LSPRs).^44,45^

Recently, some of the authors have demonstrated that plasmon-enhanced photodissociation of polluting probe molecules can be achieved by engineering self-organized plasmonic arrays.^23^ Here, the plasmonic functionalities in the arrays have been combined with the electrical transport properties in a semitransparent template, thus creating an ideal platform for developing a large-area self-organized photoelectrochemical device. To this aim, the plasmonic template is coated with a conformal TiO_2_ thin film that acts as the main catalyst medium.

Conformal ultrathin TiO_2_ films of 10 and 5 nm thickness (samples NW10 and NW5) are deposited via RF sputtering deposition under UHV conditions on two equivalent plasmonic NW devices and onto flat Au films (thickness 50 nm), acting as reference samples, respectively, called samples Ref10 and Ref5 (details in the Methods Section). The thickness of the TiO_2_ film has been chosen in the ultrathin range to be compatible with the hot-electron diffusion length in TiO_2_ and to improve coupling with the plasmonic near-field at visible frequencies.^44^

**Figure 3:**
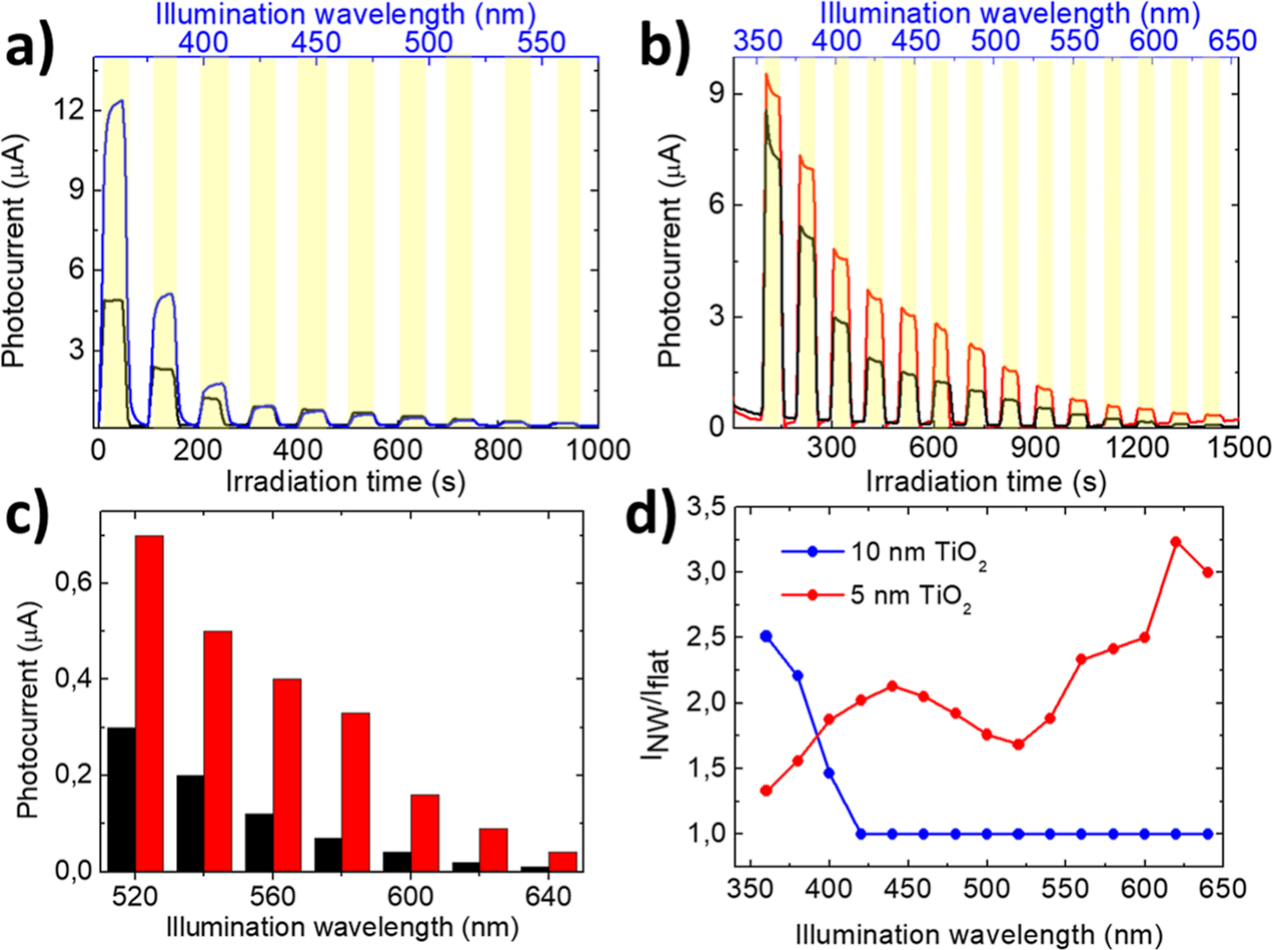
(a,b) Photoelectrochemical current detected by illuminating the samples NW10 (blue line, panel a), NW5 (red line, panel b), and their corresponding flat Au–TiO_2_ films acting as references (black lines) with an unpolarized monochromatized light source. The light is turned on for a fixed time interval (50 s, corresponding to the yellow boxes) on both the NW arrays and the reference device, increasing the wavelength from the near-UV to the visible spectrum at the 20 nm step. The illumination wavelengths are shown on the upper blue axis. (c) Extract of photoelectrochemical current measurements for illumination between 520 and 640 nm relative to the NW5 device (red bar) and its corresponding reference (black bar). (d) Plot of photocurrent gain *G* = *I*_NW_/*I*_flat_ as a function of the illuminating wavelength for samples NW10 (blue dots) and NW5 (red dots).

To measure the photoelectrochemical properties of these hybrid plasmonic/oxide devices, they are employed as electrodes in an oxygen evolution reaction (OER) induced in a NaOH solution (0.5 M) by applying a bias potential of 0.5 V to the NW device with respect to a bulk Pt counter-electrode (as schematized in Figure 2e). To evaluate the photoelectrochemical activity of the samples at different wavelengths, the photocurrent has been detected by illuminating the system with a monochromatized and unpolarized xenon lamp at different increasing wavelengths from 360 to 640 nm, at 20 nm increments. At each wavelength, steady-state excitation occurred for a duration of about 50 s, which is substantially longer than the photocurrent rise time that is in the range of a few seconds. In Figure 3a,b the photoelectrochemical current detected on the NW array devices NW10 and NW5 (blue and red curves, respectively) are compared with a corresponding flat reference Au–TiO_2_ film of the same thickness (black curves).

Figure 3a shows the response of NW10 and of the reference sample Ref10. A strong photocurrent signal, reading up to 12 μA in the NW arrays, is detected between 360 and 420 nm wavelength due to the high photoelectrochemical activity of the TiO_2_ film in this spectral range, which ensures direct photoabsorption across the oxide band gap. Remarkably, both the increase of the surface-to-volume ratio in the nanopatterned device (red curve) with respect to the flat reference sample (black curve) and enhanced reactivity in the nanostructured templates induce an amplification of the signal by a factor of about 2. Figure 3b shows the photoelectrochemical response for sample NW5 capped by a thinner TiO_2_ coating, which has been optimized to strongly couple the plasmonic hot-electrons of the Au nanoantennas with the TiO_2_ surface active sites. Under this condition, we observe a weaker effect in the near-UV range since a lower overall optical absorption occurs in the thinnest TiO_2_ film, while an amplification effect is detected in the visible spectral range. An extrapolation of the photocurrent behavior of the thinnest device in the 520 −640 nm spectral range (Figure 3c) allows us to better show the plasmonic amplification of the photocurrent for the NW sample with respect to the corresponding reference sample. Remarkably, for illumination at wavelengths higher than 600 nm, a nonzero signal is detected in the NW sample, while the reference sample shows a response equivalent to the instrumental noise level. It is also worth noting that such an extended spectral response is not detected for the NW sample capped by a 10 nm TiO_2_ film, for which the measured photocurrent is at the noise level in this spectral range (see Figure S2 for details).

The thinner NW array device shows a photoelectrochemical activity in a spectral region where the optical absorption of the TiO_2_ layer is extremely weak, below the detection limit.^46−48^ To better highlight this enhancement, we calculate the photocurrent gain as the ratio between the current detected in the NW device and the current detected in the reference sample: , for both the NW10 and the NW5 devices (Figure 3d, red and blue dots, respectively).

The NW10 sample (blue curve) shows a photocurrent response in the active spectral range of TiO_2_ that is amplified due to the larger active surface area induced by the nanopatterned templates and due to the increase of optical interaction in the laterally disconnected Au–TiO_2_ NWs. For the NW5 sample (red curve), a broadband amplification effect is detected that reaches a factor of 2.5–3 in the spectral range between 560 and 640 nm. We stress that the strongly enhanced sensitivity for wavelengths higher than 600 nm just represents a lower limit since the photocurrent of the reference Au/TiO_2_ film drops below the instrumental noise level of the setup (in the range of 39 nA).

**Figure 4:**
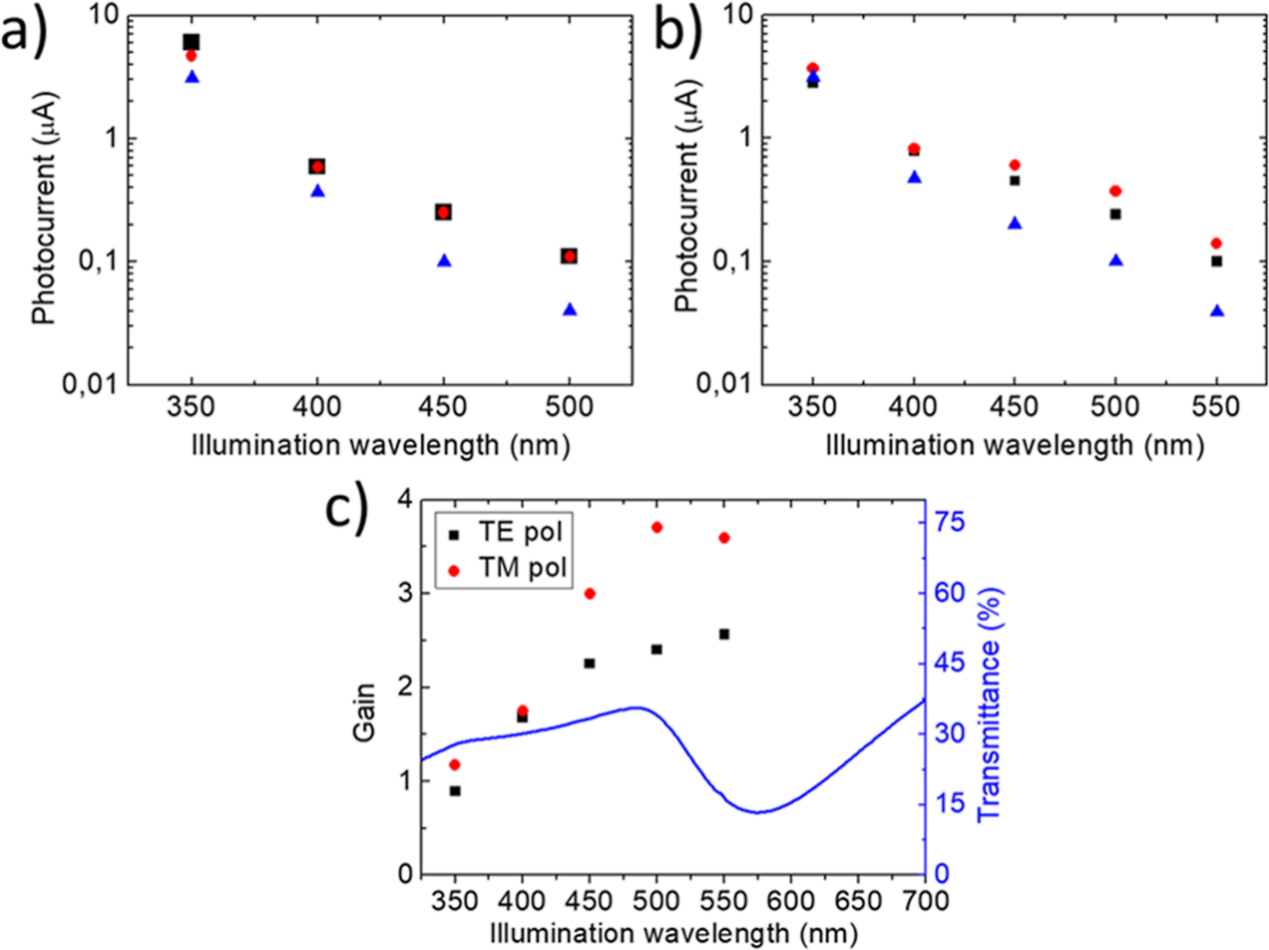
(a,b) Photocurrent measured under linearly polarized illumination at different wavelengths for samples NW10 and NW5, respectively. The black squares correspond to the Au NWs-TiO_2_ device illuminated with TE polarization, while the red dots correspond to a TM polarization (both polarizations are defined in Figure 2b). The blue triangles refer to a flat sample illuminated with polarization corresponding to a TM polarization for the Au NWs-TiO_2_ device. (c) Relative photocurrent gain for sample NW5 under polarized illumination, normalized to the Ref5 sample. The red dots correspond to a TM polarization, while the black squares correspond to a TE polarization. The blue line corresponds to the transmission spectrum of the sample with a TM polarization.

To better understand the origin of the observed amplification behavior from the anisotropic NWs that support localized plasmon resonances, we probed an equivalent set of samples with a different setup, which allows for performing photoelectrochemical measurements under polarized illumination with a monochromatic beam characterized by an optical intensity in the range of 10 mW/cm^2^, slightly lower than that used in the unpolarized experiments of Figure 3. As shown in detail in Supporting Information Table S1, different wavelengths increasing from 350 to 550 nm have been employed. Under these illumination conditions, we also characterized the signal stability by performing consecutive on–off illumination cycles, as shown in detail in Supporting Information Figure S3. Figure 4a,b shows the photocurrent values measured for different polarized illumination wavelengths on the NW10 and NW5 devices, respectively. For what concerns the NW10 device (Figure 4a), there is no substantial difference between the TE polarization (black squares) and the TM polarization (red dot); however, for both the polarizations, the NW device shows an amplified photocurrent with respect to the reference sample (Ref10 film, blue triangles). Similarly for the NW5 device (Figure 4b), a gain in the photocurrent is still visible for both polarizations with respect to the reference film. However, a dichroic behavior of the photocurrent is detected with an amplification of the signal for TM polarization with respect to the TE polarization. This response at visible frequencies suggests a crucial role played by the plasmonic excitation that is expected to promote resonant near-field amplification and hot electron generation.

To better highlight the polarization and spectral dependence of the photocurrent signal, we calculate the relative photocurrent gain as the ratio between the photocurrent measured in sample NW5 and in sample Ref5 (Figure 4c). A gain is observed for excitation wavelength higher than 350 nm for both TE and TM polarization of the incident light; however, a clear polarization dependence is observed with maximum gain detected for transversal TM polarization. Under this condition, a maximum gain of about 3.5–3.8 is detected between 500 and 550 nm wavelengths, suggesting a crucial role played by the localized plasmon excitation of the NWs which are excited in TM polarization. The low incident power provided by the experimental setup at higher wavelengths did not allow for characterizing the device in the red-shifted spectral region beyond 550 nm since the photocurrents dropped below the instrumental noise level. A direct comparison with the TM transmission spectrum of the NW device (blue line in Figure 4c) highlights an increase of the photoelectrochemical gain in the registry with the onset of interband transitions of gold (excited both for TE and TM polarization) and with the LSPRs (excited in TM polarization).

The enhancement of the photocurrent signal of the NW device, with respect to the reference flat film, can be attributed to multiple effects. The first one is the increased surface-to-volume ratio in the NW arrays, resulting in a larger active interface between the TiO_2_ films and the electrolyte solution, corresponding to a factor 1.3, that contributes to polarization- and wavelength-independent effect in all the NW devices investigated. Additionally, in the visible spectral range, we detect a polarization-dependent amplification effect that promotes photocurrent enhancement when the excitation is transversely polarized with respect to the gold NWs, suggesting the role of localized plasmon resonances supported by the Au NWs. Under these conditions, both a resonant near-field amplification and an enhanced hot-carrier generation take place, the first indirectly contributing to the photocurrent via increased local photoexcitation and the second directly promoting hot-carrier injection through the ultrathin TiO_2_ film. We stress that the hot-electron population is characterized by a relatively broad-band spectral distribution that overlaps the interband transition region, thus inducing a spectral broadening of the photocurrent with respect to the detected plasmonic mode. The diffusion length of the Au hot-electrons through the TiO_2_ interfaces is very small, in the range of a few nanometers,^49^ so the thickness of the TiO_2_ layer has to be limited in that same range. As a matter of fact, the contribution of Au hot-electrons is almost completely absent in the case of 10 nm thick TiO_2_ films (Figure 4a).

## Conclusions

In this work, we develop a large-area photoelectrochemical device based on a self-organized array of Au NWs coated with an ultrathin TiO_2_ film. The self-organized Au NW arrays are fabricated in a single maskless step over large areas (cm^2^), by combining ion-assisted nanopatterning of dielectric surfaces with glancing angle metal evaporation. The Au NW arrays act both as a transparent conductive layer and as a plasmonic medium, resonantly promoting the photoconversion efficiency of a reference photoelectrochemical OER at visible photon energies well below the TiO_2_ band gap. A spectrally selective and polarization-dependent detection of the photocurrent demonstrates the key role played by the plasmonic NWs that promote both polarization-sensitive optical absorption, via resonant near-field enhancement, and hot-carrier injection over a broader visible spectral range, which extends well beyond the band gap of the TiO_2_ layer.

These results qualify the hybrid Au NWs-TiO_2_ as a promising and scalable self-organized platform for solar energy conversion via photochemical reactions and pave the way for large-area photonics and energy storage applications.

## Methods

### Illumination Setup

All the samples are illuminated with a Newport TLS130B-300X tunable light source, equipped with a 300 W xenon arc lamp, a CS130B monochromator, and a 1-in. output flange. The monochromatized beam (±25 nm of bandwidth) passes through a Thorlabs WP25M-UB ultrabroadband wire grid polarizer before reaching the samples.

### Photoelectrochemical Measurement

For the photoelectrochemical measurement, we used a two-electrode configuration where our sample (gold NWs-TiO2 device or reference sample) acts as an electrode and a platinum plate acts as counter-electrode in a 0.5 M NaOH solution. A bias voltage of 0.5 V was applied between the two electrodes, measuring the current flowing both in the dark and under illumination. The system is contained in a Teflon photoelectrochemical cell equipped with a quartz window for sample illumination. The photoelectrochemical signal from the sample was acquired by an Ossila potentiostat T2006.

### TiO_2_ Sputtering Deposition

TiO_2_ layers were grown with a custom-made RF sputtering system using a 2 in. titanium target. The reactive RF sputtering experiment was run in a mixed argon and oxygen atmosphere at a power *P* = 10 W and sample–target distance *d* = 8.5 cm. The TiO_2_ layer thickness was monitored with a calibrated quartz microbalance.

Morphological characterization of the samples was performed by SEM (Hitachi SU3500) and by AFM (Nanosurf S Mobile) operating in contact mode.

## Supporting Information Available

The Supporting Information show detailed analysis of the nanopattern topography performed via self-correlation of the AFM image. Additionally the photoelectrochemical current plots detected by illuminating the samples NW5, NW10, Ref10, and Ref5 are shown, as well as the stability of the photocurrent signal for sample NW10 under 10 cycles of illumination (PDF)

## Abbreviations

NW5: Au nanowire arrays coated with 5 nm TiO_2_
NW10: Au nanowire arrays coated with 10 nm TiO_2_
Ref5: Flat Au film coated with 5 nm TiO_2_
Ref10: Flat Au film coated with 10 nm TiO_2_
