# Supplementary material for: Self-Organized Plasmonic Nanowire Arrays Coated with Ultrathin TiO2 Films for Photoelectrochemical Energy Storage
Source: ACS Appl Nano Mater. 2023 Nov 15;6(23):21579–86. doi: 10.1021/acsanm.3c03546 (PMC10714312; doi:10.1021/acsanm.3c03546)
Supplement: Supplementary file 1 — an3c03546_si_001.pdf [file an3c03546_si_001.pdf]

Supporting Information

Self-Organized Plasmonic Nanowire Arrays Coated with ultra-thin TiO_2 Films for Photoelectrochemical Energy Storage

Maria Caterina Giordano[1]¶, Long Duy Pham [2] ¶*, Giulio Ferrando[1]¶, Hieu Si Nguyen[2], Chi Ha Le[2], The-Hung Mai[2], Giorgio Zambito[1], Matteo Gardella[1] and Francesco Buatier de Mongeot[1]*

[1] Dipartimento di Fisica, Università di Genova, Via Dodecaneso 33, 16146 Genova (Italy)

[2] Institute of Material Science, Vietnam Academy of Science and Technology,18 Hoang Quoc Viet, Cau Giay, Hanoi (Vietnam).

In Figure S1a we show the self-correlation of the AFM image reported in the main text in Figure 1a. It was extracted through the software WsXM. The characteristic wavelength of the ripples is computed by

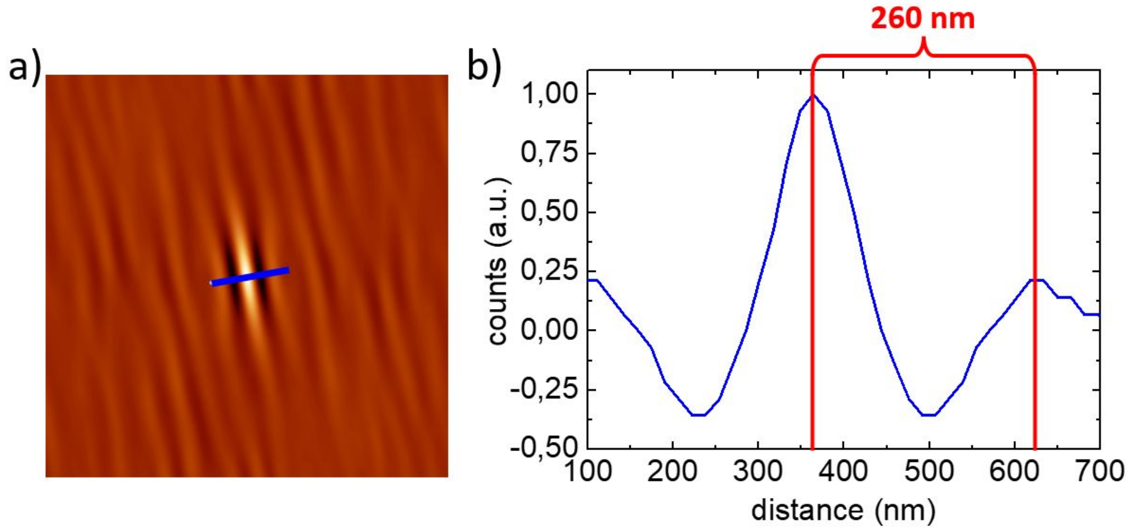

measuring the distance between the maximum and the second peak of the line profile of Figure S1b.

Figure S1 a,b) Self-correlation of the AFM image reported in Figure 1a and the corresponding cross-section profile along the blue line reported in panel a.

In Figure S2 are reported the raw data relative to the 5nm Au-TiO_2 device (black line) and the films used as reference, respectively with 5nm (blue line) and 10 nm (purple line) of TiO_2 deposited. From the image is possible to see that the signal of the nanostructured device is clearly out of the measurement noise.

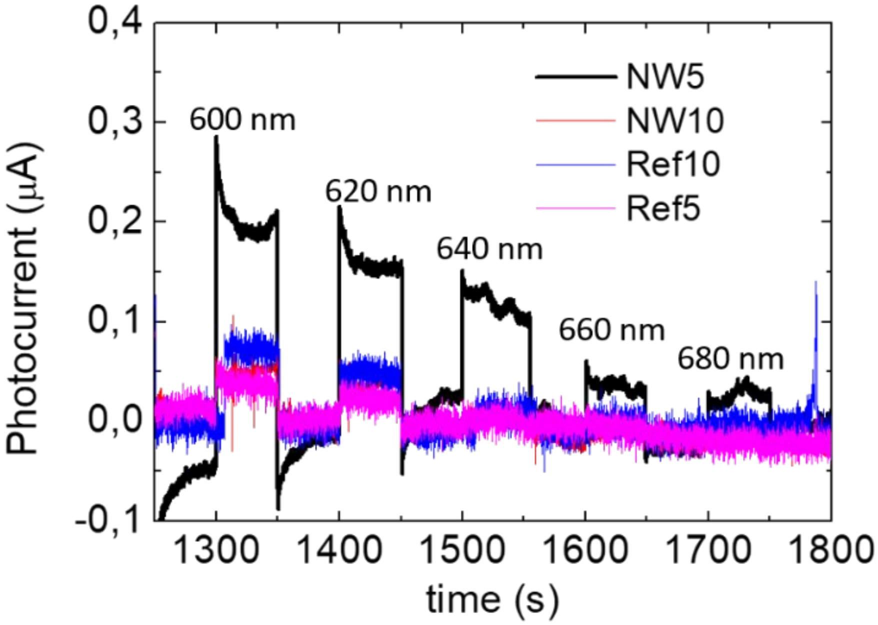

Figure S2 Photoelectrochemical current detected by illuminating the NW5 (black line), NW10 (red line), Ref10 (blue line) and Ref5 purple line).

In Table S1 are reported the power densities measured with a Newport 818-UV optical power detector connected to a Newport 1919-R optical power meter. All the power densities reported are measured at the same distance of the samples used for the experiments.

wavelength (nm)	TM (mW/cm^2)	TE (mW/cm^2)
350	10,2	10,9
400	14,1	14,4
450	17	16
500	14,8	13
550	11,4	9,9
600	9,3	7,15
650	7,3	5,15
700	3,5	6,9

Table S1 Incident power densities at different wavelength and polarization.

The stability of the photocurrent signal induced in presence of the Au NW arrays-TiO_2 sample has been characterized by illuminating the sample NW10 at 350 nm wavelength and TE polarization for 10 consecutive on-off cycles (Figure S3). We observed a relatively stable behavior with average peak to valley signal of about 5.5 μA, and a relative signal reduction of about 10% between the first and last cycle. This behavior agrees with state-of-the-art research in the field[1-3].

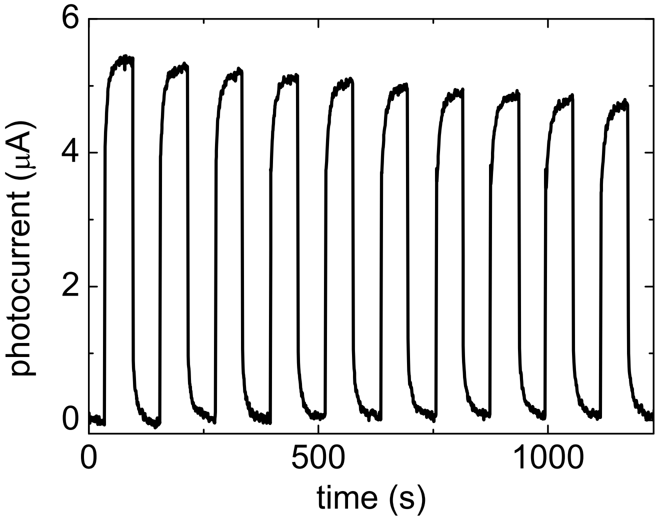

Figure S3: Photocurrent signal detected on Sample NW10 by 10 cycle illumination at 350 nm wavelength with TE polarization.

References

(1) Chen, W.; Lu, Y.; Dong, W.; Chen, Z.; Shen, M. Plasmon Mediated Visible Light Photocurrent and Photoelectrochemical Hydrogen Generation Using Au Nanoparticles/TiO2 Electrode. *Mater. Res. Bull.* **2014**, *50*, 31–35. https://doi.org/10.1016/j.materresbull.2013.10.017.

(2) Liu, S.; Xu, Y.-J. Photo-Induced Transformation Process at Gold Clusters-Semiconductor Interface: Implications for the Complexity of Gold Clusters-Based Photocatalysis. *Sci. Rep.* **2016**, *6* (1), 22742. https://doi.org/10.1038/srep22742.

(3) Song, J.; Zeng, D.; Xie, Y.; Zhang, F.; Rao, S.; Wang, F.; Zhao, J.; Zhang, J.; Wang, L. Preparation of CdS Nanoparticles-TiO2 Nanorod Hererojunction and Their High-Performance Photocatalytic Activity. *Catalysts* **2020**, *10* (4), 441. https://doi.org/10.3390/catal10040441.
